# ﻿Revision of the genus *Ninomimus* Lindberg, 1934 (Hemiptera, Lygaeoidea, Ninidae), with the description of a new species from China

**DOI:** 10.3897/zookeys.1228.140752

**Published:** 2025-02-21

**Authors:** Cuiqing Gao, Suyan Cao

**Affiliations:** 1 Co-Innovation Center for Sustainable Forestry in Southern China, College of Forestry and Grassland, Nanjing Forestry University, Nanjing, Jiangsu 210037, China Nanjing Forestry University Nanjing China

**Keywords:** Heteroptera, identification key, *Ninomimusfuscus*, taxonomy, true bugs

## Abstract

The species of *Ninomimus* Lindberg, 1934 are reviewed. A new species, *Ninomimusfuscus***sp. nov.**, is described from Hunan Province, China. It differs from its two congeners in its shiny body and translucid hemelytra. A diagnosis of the genus, a key to the included species, photographs of habitus and male genitalia of selected species, and micrographs of the pruinose area of the type species are also presented.

## ﻿Introduction

The Ninidae (Hemiptera, Heteroptera, Lygaeoidea) are a small family comprising five genera and 14 species worldwide (Slater 1964; Dellapé and Henry 2024). This family was erected by Barber (1956) as a tribe within Cyminae (Lygaeidae) and elevated to family level by Henry (1997). Prior to this study, the genus *Ninomimus* Lindberg, 1934 comprised of two species, *N.assamensis* (Distant, 1901), distributed in Japan and India, and *N.flavipes* (Matsumura, 1913), distributed in Russia, Korea, Japan, and China (Zheng and Zou 1981; Péricart 2001).

In this study, a new species, *Ninomimusfuscus* sp. nov., is identified and described from Hunan Province, China. As a result, three species are now included in the genus, with two of them occurring in China. A key to all known species of the genus is given. In addition, the ultrastructure of the cuticular pruinose area of the type species is also explored using scanning electron microscopy.

## ﻿Materials and methods

Images of the specimens were captured using a Canon EOS R6 camera mounted on a Wemacro focus stacking rail, equipped with a Laowa 25 mm f/2.8 2.5–5× Ultra Macro lens and then z-stacked using Helicon Focus v. 8.1.0. Images of the genital segments were obtained using MSHOT Image Analysis System v. 1.1.4. A female of *N.flavipes* was sputter-coated with gold and observed using a scanning electron microscope (SEM, FEI Quanta 200). Figures were post-processed with Adobe Photoshop CC 2023.

Details of male dissection methods and terminologies used in this article are those given in Ashlock (1957). All measurements in the text are given in millimeters.

Abbreviations of institutions where the material were deposited:

**IZAS** Institute of Zoology, Academia Sinica, Beijing, China

**NKUM** Institute of Entomology, Nankai University, Tianjin, China

**NJFU** Nanjing Forestry University, Nanjing, China

## ﻿Results

### ﻿ Ninomimus

Taxon classificationAnimaliaHemipteraNinidae

Lindberg, 1934

2E47480E-4423-5F71-9AD8-44D84CE26143

**Figure 1.**
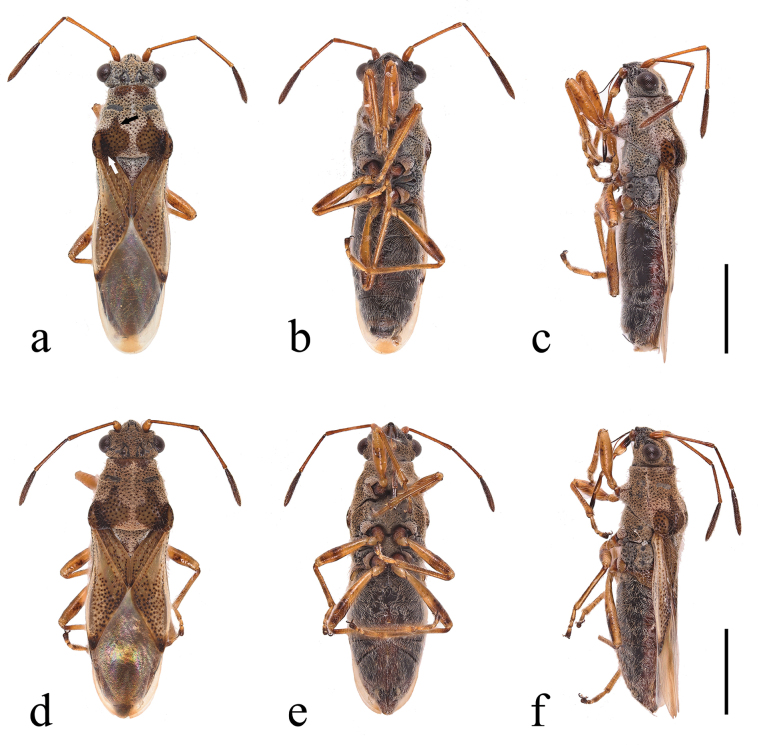
*Ninomimusflavipes* (Matsumura, 1913) **a** male in dorsal view; black arrows, indicating inner anterior margin of large spot at humeral angle pointed anteriorly; white arrows, indicating middle spot at humeral angle slightly fuscous **b, c** male in ventral and lateral views, respectively **d–f** female in dorsal, ventral, and lateral views, respectively. Scale bars: 1.0 mm.

**Figure 2.**
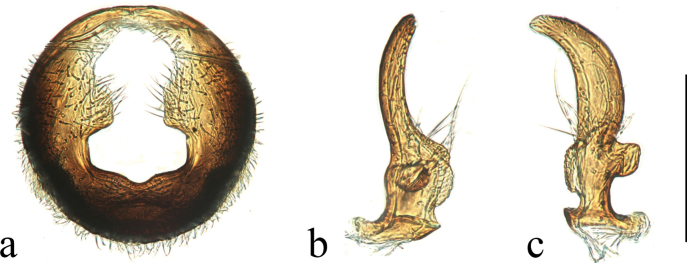
Genitalia of *Ninomimusflavipes* (Matsumura, 1913) **a** pygophore, inposterodorsal view **b, c** right paramere, in dorsal and ventral view. Scale bars: 0.2 mm.

**Figure 3.**
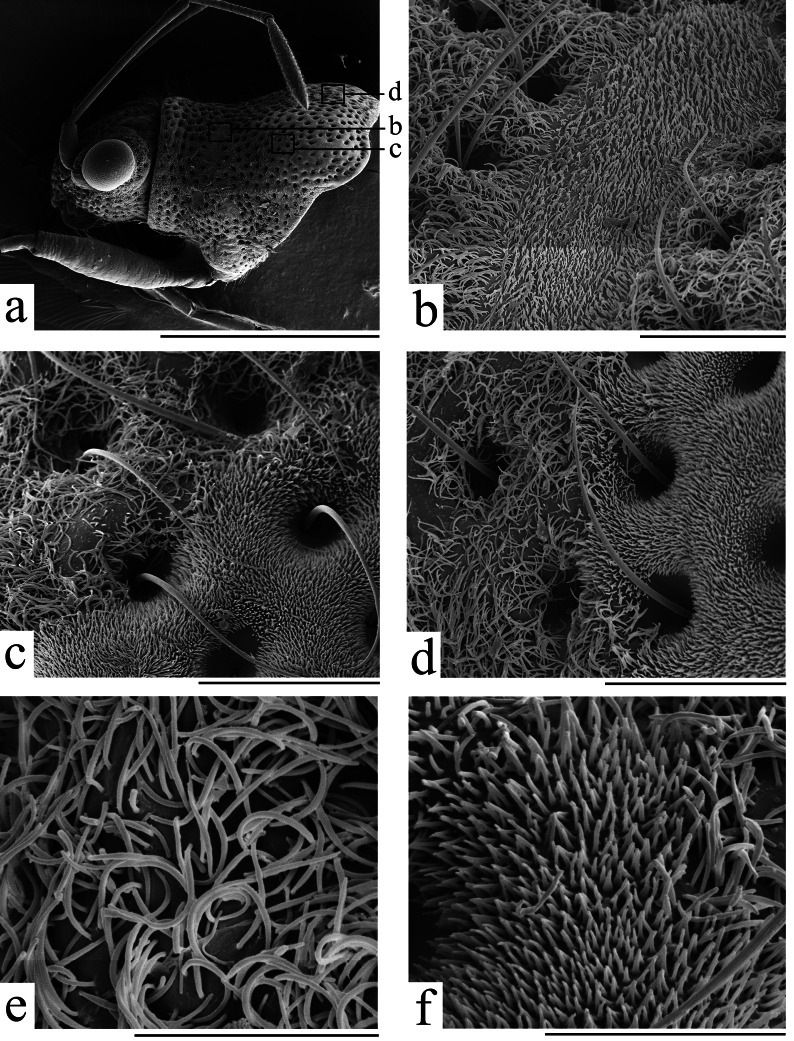
SEM micrographs of *Ninomimusflavipes* (Matsumura, 1913) **a** head and prothorax, lateral view **b** calli **c** edge of left humeral large spot **d** edge of right humeral large spot **e** dense, long, curly, hair-like microtrichia (pruinose area) **f** short, erect microtrichia (non-pruinose area). Scale bars: 1.0 mm (a); 0.05 mm (b–d); 0.02 mm (e, f).

**Figure 4.**
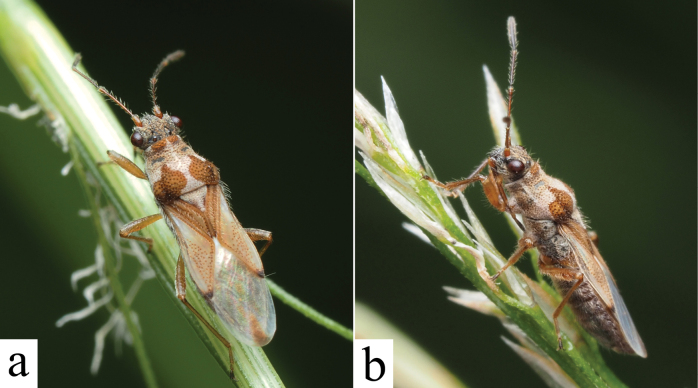
*Ninomimusflavipes* (Matsumura, 1913), live habitus. **a** dorsal view **b** lateral view.

**Figure 5.**
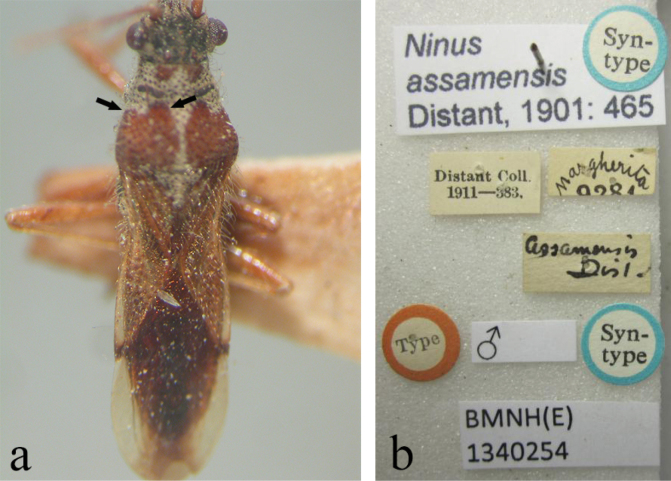
Type photographs of *Ninomimusassamensis* (Distant, 1901) **a** syntype, in dorsal view; black arrows indicate medial and lateral angles of anterior margin of spot on humeral angle bluntly extended anteriorly; **b**, labels (photographed by Előd Kondorosy).

**Figure 6.**
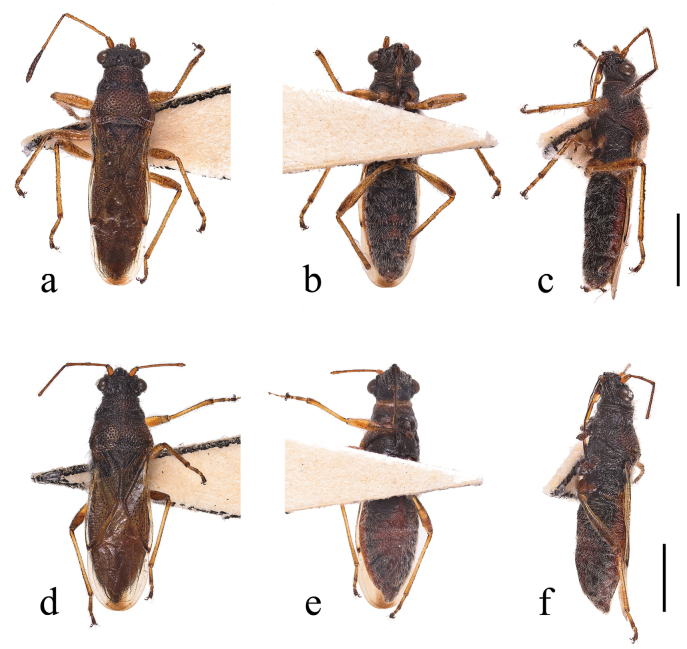
*Ninomimusfuscus* sp. nov. type photos **a–c** holotype, male, in dorsal, ventral, and lateral view, respectively **d** paratype, female, in dorsal, ventral, and lateral view, respectively. Scale bars: 1.0 mm.

**Figure 7.**
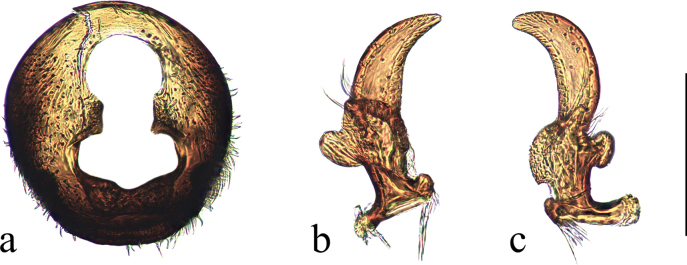
Genitalia of *Ninomimusfuscus* sp. nov. **a** pygophore, in posterodorsal view **b, c** right paramere, in dorsal and ventral view, respectively. Scale bars: 0.2 mm.

Figs 1 , 2 , 3 , 4 , 5 , 6 , 7

Ninomimus : Lindberg 1934: 8; Scudder 1957: 106–107; Stichel 1958: 318; Slater 1964: 425–426; Zheng and Zou 1981: 40–41; Péricart 2001: 70.

#### Type species.

*Ninomimuslundbladi* Lindberg, 1934 (= *N.flavipes*), by original monotypy.

#### Diagnosis.

Body elongate, nearly parallel-sided, covered with long setae. Head wider than long; eyes sessile and large; first antennal segment short and thick, with length close to eye width; second longest, third and fourth nearly equal in length; labium with apical half of first segment swollen. Pronotum slightly widened posteriorly, wider than long; humeral angles rounded; posterior margin shallowly concave before scutellum; posterolateral angle of metapleuron acute. Scutellum with tip slightly bifid. Hemelytra extending to tip of abdomen; clavus with three rows of punctures, one along of corium incomplete, the other two extending to apex. Membrane with a central longitudinal dark brown stripe. Femora slender and unarmed; posterior tarsi with first segment slightly longer than combined length of the other two.

*Ninomimus* is distinguished from *Cymoninus*, *Paraninus*, and *Neoninus* by the swollen apical half of the first labial segment. *Ninomimus* is distinguished from *Ninus* by the punctae on the apical half of the clavus and the corium, which bear a dense series of punctures extending anterior to the R+M veins as well as along these veins, whereas in *Ninus*, the apical half of the clavus is impunctate, and the corium is punctate only along the R+M veins.

### ﻿Key to species of *Ninomimus* Lindberg

**Table:** 

1	Head, scutellum and pronotum except middle of anterior margin and postero-lateral spots covered with thick white pruinosity; ground color of pronotum yellowish brown (Figs 1, 4)	**2**
–	Head, scutellum and pronotum shining, without thick white pruinosity; ground color of pronotum blackish brown (Fig. 6a, d)	***N.fuscus* sp. nov.**
2	Hemelytra opaque; membrane with longitudinal fuscous stripe at apex only; middle spot at humeral angle slightly fuscous, and inner anterior angles of large spot on humeral angle usually anteriorly pointed (Figs 1a, d, 4); second antennal segment conspicuously longer than fourth	***N.flavipes* (Matsumura, 1913)**
–	Hemelytra semitranslucid; membrane with longitudinal fuscous stripe extending along whole length of membrane and broader at middle; humeral angle not fuscous, and both medial and lateral angles of anterior margin of large spot on humeral angle bluntly extended anteriorly (Fig. 5a); second antennal segment only very slightly longer than fourth	***N.assamensis* (Distant, 1901)**

### ﻿ Ninomimus flavipes

Taxon classificationAnimaliaHemipteraNinidae

(Matsumura, 1913)

D21E0316-08D8-5CBE-B38B-4FAD500B9911

Figs 1 , 2 , 3 , 4

Lygaeosoma flavipes Matsumura, 1913: 142.
Ninomimus lundbladi Lindberg, 1934: 9.
Ninus flavipes : Esaki 1950: 223.
Cymoninus flavipes : Kormilev 1955: 4.
Ninomimus flavipes : Scudder 1957: 107; Stichel 1958: 318; Slater 1964: 426; Zheng and Zou 1981: 40–41; Péricart 2001: 70.

#### Material examined.

China – Anhui Prov. • 8 ♂ 6 ♀; Shucheng County, Wanfo Mountain; 31.0753°N, 116.5628°E; alt. 465 m; 18 May 2024; S.Y. Cao & C.Q. Gao leg.; NJFU. – Heilongjiang Prov. • 1 ♂ 1 ♀; Hailin City, Hengdaohezi Town, Weihu Mountain; 25–30 Jul. 2003; Y.L. Ke leg.; NKUM. – Jilin Prov. • 1 ♀; Erdaobaihe Town; alt. 740 m; 7 Jul. 1986; Li leg.; NKUM. – Henan Prov. • 3 ♀; Nanyang City, Huangshi’an; 17 Jul. 1998; H.F. Zhang leg.; NKUM. – Shaanxi Prov. • 1 ♂; Feng County, Qinling Railway Station; alt. 1400 m; 28 Jul. 1994; W.J. Bu leg.; NKUM. • 22 ♂ 26 ♀; Foping Nature Reserve; alt. 1100 m; 19–20 Jul. 2006; D. Ding & J.Y. Xu leg.; NKUM. – Zhejiang Prov. • 10 ♂ 10 ♀; Qingyuan County, Baishanzu; alt. 550–1650 m; 15–16 Jul. 1994; H. Wu leg.; NKUM. • 6 ♂ 13 ♀; Taishun County, Wuyanling Nature Reserve; 4–5 Aug. 2007; Z.H. Fan & W.B. Zhu leg.; NKUM. • ♀; Lin’an District, Changhua Town, Qingliangfeng Mountain; alt. 1000 m; 17 May 2012; W.B. Yin leg.; NKUM. – Hubei Prov. • 7 ♂ 6 ♀; Fang County; 14–17 Jun. 1977; L.Y. Zheng & Q. Mu leg.; NKUM. • 5 ♂ 3 ♀; Shennongjia Nature Reserve; 22 Jun.–9 Jul. 1977; L.Y. Zheng & H.G. Zou leg.; NKUM. • 13 ♂ 15 ♀; Lichuan City, Xingdou Mountain; 31 Jul. 1999; H.J. Xue leg.; NKUM. • 6 ♂ 6 ♀; Tongshan County, Jinjigu Valley; alt. 450 m; 30 Jul.–11 Aug. 2010; Y. Wang leg.; NKUM. – Jiangxi Prov. • 2 ♂; Lushan Botanical Garden; 21–25 Jul. 1957; S.H. Ying leg.; NKUM. • 3 ♀; Jiulian Mountain; 16 Jul. 2002; H.J. Xue leg.; NKUM. – Hunan Prov. • 1 ♀; Huaihua City; 20 Jul. 1995; W.J. Bu leg.; NKUM. • 10 ♂ 13 ♀; Yanling County, Taoyuandong; alt. 1000 m; 17–18 Jul. 2004; Y.L. Ke & J.Y. Xu leg.; NKUM. • 1 ♂ 3 ♀; Dong’an County, Shunhuang Mountain; alt. 1200 m; 28 Jul. 2004; Y.L. Ke leg.; NKUM. – Fujian Prov. • 2 ♂ 1 ♀; Shanghang County, Buyun Village; 6 May 1993; W.J. Bu leg.; NKUM. • 1 ♂; Dehua County, Shangyong Town, Houzhai Village; 15 Sep. 2022; W.L. Zhang leg.; NKUM. – Hainan Prov. • 6 ♂ 3 ♀; Wanning City, Jianling Nature Reserve; 28–29 Jul. 2008; C.Q. Gao, Z.H. Fan & X. Zhang leg.; NKUM. • 1 ♀; Wanning City, Xinglong District; alt. 100 m; 1 Aug. 2008; Z.H. Fan leg.; light trap; NKUM. – Guangxi Prov. • 1 ♂ 2 ♀; Longsheng Autonomous County; 25–28 Aug. 1964; L.C. Wang & S.L. Liu leg.; NKUM. • 18 ♂ 14 ♀; Mao’er Mountain; alt. 1100 m; 20 Apr. 2002; H.J. Xue leg.; NKUM. – Sichuan Prov. • 2 ♀; Dujiangyan City, Gaoyuan Village; alt. 1100 m; 15 Aug. 2011; Y. Liu leg.; NKUM. – Guizhou Prov. • 5 ♂ 7 ♀; Xishui County; 1 Jun. 2000; H.J. Xue leg.; NKUM. • 14 ♂ 18 ♀; Suiyang County, Kuankuoshui Nature Reserve; alt. 850–1700 m; 4–8 Jun. 2010; K. Dang leg.; NKUM. • ♀; Suiyang County, Kuankuoshui Nature Reserve; alt. 1300 m; 16 Aug. 2010; Y.H. Wang & X. Sun leg.; NKUM.

#### Redescription.

Body slender, covered with long, yellowish-white setae, densest at basal part of hemelytra. Head (except for tylus and a pair of large spots anterior to ocelli), pronotum (except of two spots at middle of anterior margin, calli, and a pair of large spots at humeral angles), and scutellum bearing a thick white pruinosity (Figs 1, 3, 4).

***Head***: greyish brown; eyes large and rounded; setae on third and fourth antennal segments approximately twice of diameter of respective segments (Fig. 4); labium extending to mesocoxae, yellowish brown; basal part of first segment and fourth segment blackish brown; apical swollen part of first segment light brown.

***Thorax***: pruinose area of pronotum greyish white, composed of dense, long, curly, hair-like microtrichia, as seen in the SEM micrographs (Figs 1, 3e, 4); calli black, two spots at middle of anterior margin and a pair of large spots at humeral angles brown, composed by short, erect microtrichia showed in SEM micrographs (Fig. 3b, f); middle spot at humeral angle slightly fuscous, and inner anterior angles of large spot on humeral angle usually pointed anteriorly (Figs 1a, d, 4); posterior margin of pronotum shallowly impressed anteriad of scutellum. Scutellum completely pruinose. Propleuron brown, with a large black spot on supracoxal lobes; meso- and metapleura mostly black, with supracoxal lobes and posterior margins of metapleuron yellowish brown; pro-, meso-, and metapleura covered with the same type of pruinosity as pronotum and scutellum.

***Hemelytra***: clavus and corium pale brown, nearly opaque; apices of corium and punctures on hemelytra blackish brown; clavus with three distinct rows of punctures, with outermost row not extending to apex; punctures on corium usually not spreading to exocorium.

***Abdomen***: blackish brown, covered with long setae.

***Legs***: yellowish brown, with femora and distal tarsal segment darker.

***Male genitalia*** (Fig. 2): pygophore covered with setae; apical part of dorsal opening with anterior margin rounded rhomboid; distal margin of lateral lobe parallel; and basal part nearly rectangular with a median indentation broadly rounded on posterior margin. Paramere with both dorsal and ventral lobes broadened, rectangular; middle part of blade not apparently broadened and tip of blade rounded.

***Measurements*** (in mm; male / female, *n* = 6). Body length 3.28–3.46 / 3.37–3.53. Head length 0.39–0.40 / 0.40–0.42; width across eyes 0.78–0.85 / 0.81–0.92; eye length 0.13–0.14 / 0.13–0.15; eye width 0.20–0.22 / 0.21–0.22; eye–ocellus space 0.11–0.12 / 0.12–0.14; interocular space 0.44–0.47 / 0.45–0.48; interocellar space 0.12–0.13 / 0.12–0.13; length of antennal segments I–IV respectively 0.21–0.22 / 0.21–0.23, 0.67–0.70 / 0.68–0.73, 0.61–0.62 / 0.62–0.63, 0.66–0.67 / 0.66–0.67; length of labial segments respectively 0.31–0.32 / 0.32–0.33, 0.27–0.27 / 0.27–0.28, 0.23–0.24 / 0.24–0.25, 0.24–0.25 / 0.25–0.27. Pronotum length 0.71–0.75 / 0.74–0.79; width of anterior margin 0.51–0.54 / 0.53–0.56; width of posterior margin 0.80–0.83 / 0.82–0.88; scutellar length 0.28–0.30 / 0.30–0.34; scutellar width 0.40–0.41 / 0.40–0.42. Length of hemelytra 2.13–2.20 / 2.19–2.29; length of corium 1.39–1.43 / 1.40–1.47; length of claval commissure 0.30–0.31 / 0.33–0.34; distance of apex of clavus–apex of corium 0.67–0.69 / 0.70–0.73; distance of apex of corium–apex of membrane 0.72–0.76 / 0.76–0.79.

#### Distribution.

China (Heilongjiang, Jilin, Henan, Shaanxi, Anhui, Zhejiang, Hubei, Jiangxi, Hunan, Fujian, Hainan, Guangxi, Sichuan, Guizhou); Russia (Vladivostok); Korea; Japan (Péricart 2001).

#### Remarks.

This species is characterized by the dense, white pruinosity on its head, pronotum, and scutellum (Scudder 1957; Zheng and Zou 1981). After observing it under SEM, we found that the pruinose areas are composed of dense, long, curly, hair-like microtrichia (Fig. 3e). The non-pruinose areas are also not completely smooth, and they are in fact covered with short, erect microtrichia (Fig. 3f).

### ﻿ Ninomimus assamensis

Taxon classificationAnimaliaHemipteraNinidae

(Distant, 1901)

8CC97B4D-D83E-5E80-8BED-2A344CF2475E

Fig. 5

Ninus assamensis Distant, 1901: 465.
Cymoninus assamensis : Bergroth 1921: 168.
Ninomimus assamensis : Scudder 1957: 107–108; Stichel 1958: 318; Slater 1964: 425–426; Péricart 2001: 70.

#### Type material examined.

Syntype: India • ♂; *Ninusassamensis* Distant, 1901: 465 [printed]; Distant Coll., 1911-383 [printed]; Margherita [handwritten]; assamensis Dist. [handwritten]; Type [label with red ring, printed]; BMNH(E), 1340254 [printed]. We examined the type photo (Fig. 5b).

#### Diagnosis

(modified from Scudder 1957). Head black; antennae yellowish brown, with fourth segment darkest; second antennal segment longest; labium yellowish brown, with dark brown tip. Pronotum yellowish brown with black calli, entirely pruinose except for two spots near middle of anterior margin and a pair of large spots covering most of hind lobes and including extreme lateral areas of posterior margin; humeral angles not fuscous, and both inner and lateral angles of anterior margin of spots on humeral angles bluntly extended anteriorly (Fig. 5a). Hemelytra pale brown with darker punctures; apices of clavus and corium dark brown; apical half of clavus semitranslucid; membrane with a longitudinal brown stripe running along entire length and broader in middle.

#### Distribution.

Japan; India.

### ﻿ Ninomimus fuscus

Taxon classificationAnimaliaHemipteraNinidae

Gao & Cao sp. nov.

7BECEB69-E050-5373-B47B-7F2848D67FBF

https://zoobank.org/7A8D0D6E-F45C-4C6C-A4C9-CE4FC7B6CF10

Figs 6 , 7

#### Type material.

***Holotype***: China • ♂; Hunan Prov., Chengbu County, Dankou Town, Taiping Village; 26.3495°N, 110.2397°E; alt. 479 m; 19 Nov. 2017; Kaidong Zhao leg.; IOZ(E)1429746, IZAS. ***Paratype***: ♀; same collection data as for holotype; IOZ(E)1429745. All type specimens are deposited at IZAS.

#### Diagnosis.

Body shiny, blackish brown, without thick, dense, white pruinosity, and pruinosity poorly visible; body more elongate than its congeners, with body length-to-width ratio across eyes reaching 4.6; hemelytra translucid; pygophore with anterior margin of dorsal opening rounded; paramere with base of dorsal lobe slightly constricted, and tip of blade sharp.

#### Description.

Body shiny, without thick, dense, white pruinosity (Fig. 6).

***Head***: black, covered with long, sparse setae; wider than long, slightly declivent; eyes large and sessile; antennae yellowish brown, with fourth segment dark brown; first segment stout, second and third segments slender, fourth segment spindle-shaped; antennal ratios I < III < IV < II; bucculae short, only extending to base of antenniferous tubercles; labium reaching mesocoxae, yellowish brown, with basal part of first segment and fourth segment blackish brown, apical part of first segment prominently swollen and light yellowish brown.

***Thorax***: pronotum shiny, blackish brown, coarsely punctate except at calli and humeral angles, wider than long; lateral margins slightly convex in anterior half; posterior margin shallowly impressed anteriad of scutellum; humeral angles rounded; posterolateral angle of metapleuron acute. Scutellum blackish brown, with slightly darker margins; triangular, length equal to claval commissure; tip slightly bifid. Pro-, meso-, and metasterna blackish brown; pro-, meso-, and metapleura blackish brown except posterior margin of metapleura yellowish brown; supracoxal lobes yellowish brown.

***Hemelytra***: translucid, extending to tip of abdomen and fully covering abdominal connexivum; constricted at base; inner margin of clavus, basal half and apex of corium blackish brown; clavus with three rows of punctures, one adjacent to scutellum extending along commissure, the other two near suture margin with inner row extending to apex; corium with punctures along suture and apical margins and irregular punctures along R vein, scattered across disc to apex of R+M; membrane with longitudinal dark brown stripe extending from middle to apex, basal half of the stripe thin, widening to form a triangle towards apex.

***Abdomen***: blackish brown, with semi-erect and decumbent setae.

***Legs***: yellowish brown, covered with long setae; femora slender and unarmed; first segment of posterior tarsus longer than combined length of distal two segments; distal tarsal segment blackish brown.

***Male genitalia*** (Fig. 7): pygophore covered with suberect setae; apical part of dorsal pygophore opening with anterior margin rounded; distal margin of lateral lobe parallel; and basal part nearly rectangular with a median indentation broadly rounded on posterior margin. Paramere with both dorsal and ventral lobes broadened, with base of dorsal lobe slightly constricted; middle part of blade not apparently broadened and tip of blade sharp.

***Measurements*** (in mm, holotype ♂ / paratype ♀). Body length 3.34 / 3.59. Head length 0.41 / 0.53, width across eyes 0.73 / 0.77; eye length 0.15 / 0.16; eye width 0.20 / 0.22; eye–ocellus space 0.11 / 0.12; interocular space 0.44 / 0.44; interocellar space 0.12 / 0.13; length of antennal segments I–IV respectively 0.18 / 0.17, 0.65 / 0.59, 0.57 / 0.52, 0.61 / ?; length of labial segments respectively 0.39 / 0.39, 0.32 / 0.33, 0.25 / 0.26, 0.25 / 0.27. Pronotum length 0.78 / 0.82; width of anterior margin 0.50 / 0.54; width of posterior margin 0.87 / 0.89; scutellar length 0.30 / 0.31; scutellar width 0.37 / 0.41. Length of hemelytra 2.17 / 2.33; length of corium 1.39 / 1.45; length of claval commissure 0.36 / 0.42; distance of apex of clavus–apex of corium 0.71 / 0.79; distance of apex of corium–apex of membrane 0.80 / 0.87.

#### Etymology.

The species epithet, *fuscus*, is Latin meaning “brown” and is in reference to the new species’ dark-brown body colour, without the thick, dense white pruinosity of its congeners.

#### Distribution.

Only known from the type locality.

#### Remarks.

*Ninomimusfuscus* sp. nov. can be easily distinguished from its two congeners by its shiny and overall blackish-brown body, without a thick white pruinosity, and its clavus and corium completely translucid, while the other *Ninomimus* species show dense, thick, white pruinosity patterns, and the hemelytra are nearly opaque. The body of the new species is more slender than that of its congeners, with a body length-to-width across the eyes ratio exceeding 4.6, while this ratio in *N.flavipes* is < 4.2 and in *N.assamensis* it is approximately 4.4. The pygophore of *N.fuscus* has a rounded anterior margin of the dorsal opening, not rhomboid as in *N.flavipes*; the paramere of *N.fuscus* has the base of the dorsal lobe slightly constricted, and the tip of the blade is sharp, which contrasts with the less constricted dorsal lobe base and rounded blade tip in *N.flavipes*. Since we only examined the photographs of the type of *N.assamensis*, we were unable to compare its male genitalia morphology with those of the other species.

## Supplementary Material

XML Treatment for Ninomimus

XML Treatment for Ninomimus flavipes

XML Treatment for Ninomimus assamensis

XML Treatment for Ninomimus fuscus

## ﻿Additional information

### Author ORCIDs

Cuiqing Gao "Pseudopyrochroa">P. https://orcid.org/0000-0002-0177-5161

Suyan Cao "Pseudopyrochroa">P. https://orcid.org/0009-0008-4432-234X

### Data availability

All of the data that support the findings of this study are available in the main text.
